# Does mothers’ and caregivers' access to information on their child’s vaccination card impact the timing of their child’s measles vaccination in Uganda?

**DOI:** 10.1186/s12889-022-13113-z

**Published:** 2022-04-26

**Authors:** Bridget C. Griffith, Sarah E. Cusick, Kelly M. Searle, Diana M. Negoescu, Nicole E. Basta, Cecily Banura

**Affiliations:** 1 Department of Epidemiology, Biostatistics, and Occupational Health, McGill University Faculty of Medicine and Health Sciences, 2001 McGill College, Suite 1200, QC H3A 1G1 Montreal, Canada; 2 Division of Epidemiology and Community Health, University of Minnesota School of Public Health, Minneapolis, MN USA; 3 Department of Pediatrics, University of Minnesota Medical School Twin Cities, Minneapolis, MN USA; 4 Department of Industrial and Systems Engineering, University of Minnesota College of Science and Engineering, Minneapolis, MN USA; 5 Child Health and Development Centre, School of Medicine, Makerere University, Kampala, Uganda

**Keywords:** Child health, Immunisation, Public health, Measles, Cross-sectional survey

## Abstract

**Introduction:**

On-time measles vaccination is essential for preventing measles infection among children as early in life as possible, especially in areas where measles outbreaks occur frequently. Characterizing the timing of routine measles vaccination (MCV1) among children and identifying risk factors for delayed measles vaccination is important for addressing barriers to recommended childhood vaccination and increasing on-time MCV1 coverage. We aim to assess the timing of children's MCV1 vaccination and to investigate the association between demographic and healthcare factors, mothers'/caregivers' ability to identify information on their child’s vaccination card, and achieving on-time (vs. delayed) MCV1 vaccination.

**Methods:**

We conducted a population-based, door-to-door survey in Kampala, Uganda, from June–August of 2019. We surveyed mothers/caregivers of children aged one to five years to determine how familiar they were with their child’s vaccination card and to determine their child’s MCV1 vaccination status and timing. We assessed the proportion of children vaccinated for MCV1 on-time and delayed, and we evaluated the association between mothers'/caregivers' ability to identify key pieces of information (child’s birth date, sex, and MCV1 date) on their child’s vaccination card and achieving on-time MCV1 vaccination.

**Results:**

Of the 999 mothers/caregivers enrolled, the median age was 27 years (17–50), and median child age was 29 months (12–72). Information on vaccination status was available for 66.0% (*n* = 659) of children. Of those who had documentation of MCV1 vaccination (*n* = 475), less than half (46.5%; *n* = 221) achieved on-time MCV1 vaccination and 53.5% (*n* = 254) were delayed. We found that only 47.9% (*n* = 264) of the 551 mothers/caregivers who were asked to identify key pieces of information on their child's vaccination card were able to identify the information, but ability to identify the key pieces of information on the card was not independently associated with achieving on-time MCV1 vaccination.

**Conclusion:**

Mothers'/caregivers' ability to identify key pieces of information on their child’s vaccination card was not associated with achieving on-time MCV1 vaccination. Further research can shed light on interventions that may prompt or remind mothers/caregivers of the time and age when their child is due for measles vaccine to increase the chance of the child receiving it at the recommended time.

**Supplementary Information:**

The online version contains supplementary material available at 10.1186/s12889-022-13113-z.

## Introduction

Measles is a highly contagious disease caused by *Measles morbillivirus* (MeV); it was responsible for millions of deaths worldwide annually before the introduction of measles vaccines [1]. Even with the availability of safe and effective vaccines, measles remains an important cause of death among young children globally, especially in low- and middle-income countries (LMICs), where measles has yet to be eliminated [2]. Although there has been marked reduction of measles-associated mortality worldwide over the past several decades, the World Health Organization (WHO) African Region (AFRO) continues to report the highest measles incidence of any region, with 118 cases per one million people, and the highest incidence of measles-related deaths of any region, with 52,600 deaths reported in 2018 [3].

In Uganda, at the time of this study, the recommended measles vaccination was one dose at nine months of age, referred to as measles-containing vaccine 1 (MCV1). Delayed immunization is a strong risk factor for disease, because it leads to children having little to no immune protection via measles-containing vaccine (MCV) against measles infection after the waning of maternally acquired antibodies [4, 5]. An analysis of the timing of measles vaccination in Uganda found that the median delay in the administration of MCV1 was 2.7 weeks, but with an interquartile range (IQR) of 9.6 weeks, indicating a wide distribution in the number of weeks MCV1 was delayed [6].

Despite a steady improvement in Uganda’s measles vaccination coverage from an estimated 70% (2008) to 87% (2019) of children 12–23 months of age, outbreaks of measles remain common in both urban and rural settings [7–9]. The occurrence of these outbreaks, despite relatively high overall vaccination coverage, is attributed to a high proportion of susceptible children clustered within geographical areas, due to heterogeneity in vaccination coverage [10–12].

The degree to which delayed vaccination may contribute to epidemiologic trends in measles-endemic areas is not known. Estimating the prevalence of delayed measles vaccination, the amount of time vaccination is delayed, and elucidating factors associated with risk of delayed measles vaccination is one of the important steps toward addressing barriers to vaccination and improving on-time measles vaccination coverage.

Routine infant vaccination is available at government health facilities, private health facilities, and outreach posts within communities at specific times during the week throughout the year in Uganda. Mothers or other female caregivers are primarily responsible for ensuring that their children are vaccinated for measles at the recommended time [13–15]. Mothers/caregivers bring their child to the health facility, along with the child’s Uganda Ministry of Health Child Health Card (UCHC) or other vaccination documentation, and wait for their child's turn to be vaccinated. 

Based on the Uganda National Expanded Program on Immunisation (UNEPI)-recommended infant vaccination schedule, children are recommended to receive pneumococcal conjugate vaccine (PCV), diphtheria/tetanus/pertussis/*Hemophilus influenzae*/hepatitis B vaccine (DTwPHibHepB), and inactivated polio vaccine (IPV) at 14 weeks of age; then five and a half months later, they are recommended to receive MCV1 at nine months of age [14]. At the 14-week visit, healthcare workers overseeing childhood vaccinations are trained to verbally inform the mother/caregiver about the date to return for their child's MCV1. In this situation, the child’s vaccination document is meant to serve as a guide to let mothers/caregivers know when their child is due for their next vaccine, and this is likely the only reminder that they receive about when their child is due [16–18]. In addition, the MCV1 vaccination at nine months does not coincide with other routine health visits, which may further reduce the chance that mothers/caregivers receive any other prompts besides the age and date on the vaccination card that would remind them of when their child is due for MCV1. In some contexts, children may receive MCV before nine months of age; this is common in settings where there is an ongoing measles outbreak. If children receive MCV before nine months of age, this is noted as measles-containing vaccine 0 (MCV0) in the child’s UCHC, and mothers/caregivers are still advised to bring the child for MCV1 when they reach nine months.

In addition to routine vaccination, MCV is accessible via non-routine immunization campaigns during periods of high transmission. During these campaigns, teams of healthcare workers set up vaccination service delivery posts across the country to vaccinate children with MCV from six months to 15 years of age. These campaigns are meant to supplement, but not replace, routine vaccination [19, 20].

Children’s UCHCs are typically issued at birth, if the child was born in a health facility. If a child is born outside the health facility, the UCHC is issued the first time the child is brought for healthcare. In both cases, mothers/caregivers are instructed to retain the UCHCs until the child reaches six years of age. These cards are a record of a child’s health status from birth, including deworming and Vitamin A supplementation, growth monitoring, and immunization. Despite the importance of these cards, they are sometimes not retained until the recommended age, or they are lost or damaged [21]. In previous studies in Uganda, the possession of a UCHC was associated with childhood vaccination completion [22].

These UCHCs are often the only reminders to mothers/caregivers about upcoming childhood vaccines. It is not known whether vaccination cards are an effective method for conveying this information and whether mothers/caregivers use their child’s UCHCs for this purpose. Parental knowledge of the contents of the UCHC has been assessed in similar settings, with one study finding that parental knowledge of the timing of MCV1 increased with possession of a vaccination card [23].

The relationship between ability to identify information on the UCHC and achieving on-time MCV1 vaccination for their child is unclear. Understanding if and how mothers/caregivers locate vaccination information on their child's UCHC is important for determining if the card serves as a reminder for when a child is due for vaccination, and if that results in a child being vaccinated on-time. In this study, our primary aims are to 1) assess the proportion of children who were vaccinated with MCV1 on-time and delayed and 2) investigate the association between demographic factors, ability to identify key pieces of information on the child’s UCHC, and on-time MCV1 vaccination (vs. delayed). Our secondary aims are to 1) investigate the association between demographic and healthcare factors and mothers'/caregivers' ability to identify key pieces of information on the UCHC (vs. not being able to) and 2) investigate the association between demographic and healthcare factors and retaining the UCHC (vs. not retaining). Estimating the proportion of delayed MCV1 vaccination and assessing factors potentially associated with delayed MCV1 vaccination is an important step toward addressing and eliminating barriers to on-time vaccination.

## Methods

### Study design

We conducted a population-based, cross-sectional, door-to-door survey in Rubaga Division’s high-density, low-resource informal settlements, located in Kampala district of Uganda. Surveys were administered from June to August 2019.

### Study area

**Fig. 1:**
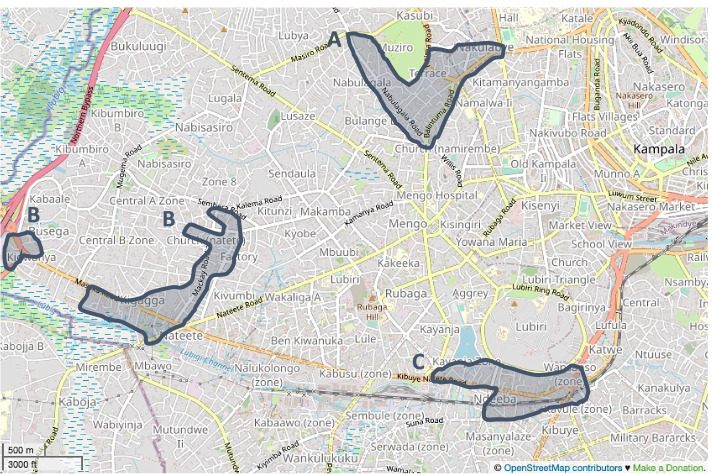
© OpenStreetMap Contributors. OpenStreetMap 2022 [24]. The three parishes that were selected for sampling are: Nakulabye (Area A); Busega (Area B); and Ndeeba (Area C)

Rubaga Division is one of the five sub-counties of Kampala district. It comprises 14 informal settlements spread throughout its 13 parishes. Based on the 2014 Uganda National Population Census, we selected three Parishes containing large informal settlements: Nakulabye, Busega, and Ndeeba. Nakulabye (Fig. 1, Area A) has an estimated 8,000 households, spread throughout its nine villages (also referred to as zones in urban settings); Busega (Fig. 1, Area B) has an estimated 6,000 households, spread throughout its nine villages; and Ndeeba (Fig. 1, Area C) has an estimated 8,000 households, spread throughout its 15 villages (Fig. 1).

We designated Local Council 1 areas (LC1s) as the study administrative unit (AU). LC1s are the smallest political-administrative unit in Uganda; in urban areas, they are comprised of multiple geographically adjacent villages. Prior to the survey administration, the study team approached community leaders to obtain necessary permissions and ask them to identify a local guide familiar with the boundaries of the selected AU. Each LC1 within an informal settlement has clearly demarcated boundaries.

Next, the study team leaders, accompanied by a local guide, conducted a household census by AU. The purpose was to enumerate and mark all households with a serial number for easy identification within the AU. Using the household census enumeration list as a sampling frame, study team leaders established a sampling interval and then randomly selected 45 potential households, which were then visited by the study team for eligibility screening of mothers/caregivers. A household was defined as a group of individuals who live under the same roof and eat from the same cooking pot [25]. If there was no eligible mother/caregiver in the selected household, the study team members visited the next household. If the mother/caregiver was away at the time of eligibility screening, the study team member returned to that household at least twice before visiting the next household. This process was repeated in each AU until the sample size was achieved.

### Participant eligibility screening and selection

Trained study staff approached each household and asked to speak with the mother/caregiver of the household. If more than one mother/caregiver was identified in the enumeration step, study staff screened all for eligibility. Potential participants were eligible if they were the mother/caregiver of a child aged one to five years of age (defined as the child had not yet reached their sixth birthday) at the time of the survey, a resident of Kampala district for more than six months during the past year, a current resident of a household in Rubaga Division, and able to understand spoken Luganda or English. If more than one mother/caregiver in a household was eligible, one was selected for inclusion via an anonymized random selection method.

### Sample size

As our primary aim was to determine the proportion of children who were vaccinated on-time among all vaccinated children, we calculated the minimum sample size necessary, assuming that 50% of vaccinated children would be vaccinated on-time and with the desire to estimate the value within plus or minus five percentage points. With an alpha of 0.05, we would need to sample 383 vaccinated children to achieve the desired power. Assuming 50% of participants would have their child’s vaccination card, based on a study in a similar setting [16], and 80% of those children would be vaccinated, we increased to a target sample size of 1000.

### Survey administration

A study staff member informed eligible participants of the objectives of the study and study procedures and invited them to participate. Next, the study staff member asked the participant if their preferred language was English or Luganda and if they could read in that language. For those who confirmed that they could read in their preferred language, they were given the informed consent form to read. For those who indicated that they were unable to read or write in English or Luganda, the study staff member read them the informed consent form in the presence of a witness. The study staff member gave participants the opportunity to ask any questions, and then the participant signed two copies of the informed consent form, if they were able, or they provided a thumbprint and their witness signed two copies of the form. One copy was retained by the study staff member, and the participant kept the other copy.

A study staff member immediately administered a 96-question survey orally to consenting participants. The interviewing study staff member recorded participant responses on a handheld tablet computer, using a series of customized REDCap questionnaire forms [26, 27]. Because the survey asked questions about the participant and their child, participants were instructed to answer all questions with respect to their child who most recently celebrated their first birthday and had not yet celebrated their sixth birthday (the index child), even if they had other children between their first and sixth birthdays. The survey took approximately 50 min to complete, on average. Upon completion of the survey, participants were given a hygiene kit to thank them for their time.

### Survey content

The survey captured demographics of the mother/caregiver and index child, mother's/caregiver's past healthcare seeking behaviour, including who in their household made decisions about the index child’s medical care, the number of antenatal care visits during their pregnancy with the index child, and the place of birth of the index child.

The survey included a section where the study staff requested permission to view and take a photograph of the vaccine-related information on the index child’s UCHC. If a child’s UCHC was not available, participants were asked to present any other documentation that included the child’s dates of vaccination, and study staff applied the same procedures. All vaccination records are referred to as the child’s vaccination card in the sections that follow.

#### Identification of information on the child’s vaccination card

**Fig. 2:**
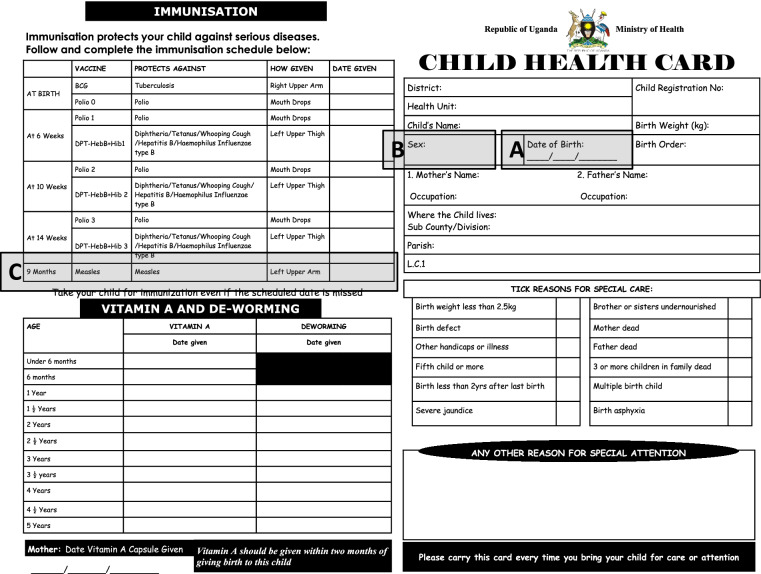
Two pages of the Uganda Ministry of Health Child Health Card (UCHC). These pages include key pieces of information the participants were asked to point to in the survey: Child’s date of birth (Item **A**); Child’s sex (Item **B**); and Information on child’s MCV1 (Item **C**), including date given

Study staff asked participants who presented a UCHC or other official documentation of vaccination that contained the index child’s basic information to identify information on their child’s card by pointing to the line where the following information was located on the card: the child’s date of birth (Fig. 2, Item A), child’s sex (Fig. 2, Item B), and date of measles vaccination (Fig. 2, Item C). Study staff categorized participants’ answers as either “correct” or “incorrect”, based on whether the mother/caregiver could locate and identify each piece of information.

### Data management

We designed and administered the surveys using the REDCap electronic data capture software Versions 9.1.2 and 9.2 [26, 27]. Study staff reviewed and entered the date of MCV from the photograph of the vaccination cards into a form created in REDCap [26, 27]. Vaccination data were double entered, compared, and any discrepancies resolved before being merged into the survey database via a unique participant identifier.

### Analysis

We used Stata 16 for data management and analysis of survey data, including calculating summary statistics and regression modelling [28]. We used R version 4.1.2 [29] and ggplot [30] to create OR plots of the model output. We considered *p*-values ≤0.05 to be statistically significant. Participants with nonmissing information were included in the final versions of each model.

#### Primary aim 1: determining the proportion of children who received MCV1 on-time vs. delayed

We first calculated descriptive statistics of demographic and healthcare characteristics of both mothers/caregivers and index children. To estimate the child’s age at time of receiving MCV, we subtracted the index child’s month and year of birth, reported by the participant, from the month and year of MCV vaccination, which we abstracted from the vaccination card. To calculate the child’s age at the time of the survey, we subtracted the date of the survey from their date of birth. Index children who were missing information about their month and year of birth in the survey or the date of MCV vaccination were excluded from the primary aim 1 analysis. We considered index children to have received MCV1 on-time if they were nine months of age at the time of MCVvaccination,to have received MCV1 delayed if they were ten months of age or older at the time of MCV vaccination, or to have received MCV early (received MCV0) if they were younger than nine months at the time of MCV vaccination. Index children who were vaccinated early were not included in the analysis of on-time MCV1 vaccination vs. delayed MCV1 vaccination. We used a one-sample test of equality of proportions with a confidence level of 0.95 to determine if there was a significant difference in the proportion of children vaccinated on-time, compared to the hypothesized proportion of 50%. We conducted sensitivity analyses to compare demographic and other characteristics of card retention using chi-square tests.

#### Primary aim 2: evaluating the association between mothers'/caregivers' and index children's demographic factors, healthcare factors, ability to identify information on the child’s vaccination card, and achieving on-time MCV1 vaccination

To determine the participants’ ability to identify information (index child’s date of birth, sex, and date of MCV1) on the index child’s vaccination card, we created a new dichotomous variable from the three responses : the participant is able to identify all three key pieces of information on the document vs. they are able to identify fewer than three or none.

Using univariate logistic regression, we evaluated the association between mothers'/caregivers' and index children’s demographic factors, health care factors, ability to identify information on the child’s vaccination card as independent variables and achieving on-time MCV1 vaccination, compared to delayed MCV1 vaccination, as the dependent variable. We computed crude odds ratios (cORs) with corresponding 95% CIs and *p*-values. Factors from these univariate models with *p* < 0.2 (mother/caregiver age, employment status, education, index child’s birth order, index child age, and index child sex) were included in an unconditional multivariable logistic regression model in which achieving on-time MCV1 vaccination (vs. delayed) was the dependent variable. We computed adjusted odds ratios (aORs) with corresponding 95% CIs and *p*-values.

#### Secondary aim 1: factors associated with ability to identify information on the child’s vaccination card

Using univariate logistic regression, we evaluated the association between mother/caregiver and index children’s demographic factors and health care factors as independent variables and ability to identify information on the child’s vaccination card as the dependent variable (defined as being able to identify three pieces of information on the index child’s vaccination card vs. not able to identify all three). We computed cORs with corresponding 95% CIs and *p*-values. Factors from these univariate models with *p* < 0.2 (who decides medical care for the child, mother/caregiver age, tribe, education, relationship to index child’s father, index child’s birth order, index child age, and index child sex) were included in an unconditional multivariable logistic regression model in which ability to identify information on the child’s vaccination card is the dependent variable. We report graphically the aOR and 95% CI for each covariate included in the full model, and cORs and aORs in Supplementary Table 1.

#### Secondary aim 2: factors associated with child's vaccination card retention

Using univariate logistic regression, we evaluated the association between mothers'/caregivers' and index children’s demographic factors and health care factors as independent variables and retention of the index child’s vaccination card, compared to not retaining the card, as the dependent variable. We computed cORs with corresponding 95% CIs and *p*-values. Factors from these univariate models with *p* < 0.2 (moved to Rubaga in the index child’s lifetime, mother/caregiver age, tribe, employment, education, index child’s birth order, index child age, index child sex, index child’s place of birth, and who decided medical care for the index child) were included in an unconditional multivariable logistic regression model in which retention of the index child’s vaccination card is the dependent variable. We report graphically the aOR and 95% CI for each covariate included in the full model, and cORs and aORs in Supplementary Table 2.

### Ethical review

This study was reviewed and approved by the Makerere University School of Medicine Research and Ethics Committee (SOMREC) (Study number: 2018–117), the Uganda National Council for Science and Technology (UNCST), and the University of Minnesota Institutional Review Board (Study number: STUDY00004955).

## Results

### Participant characteristics

**Fig. 3:**
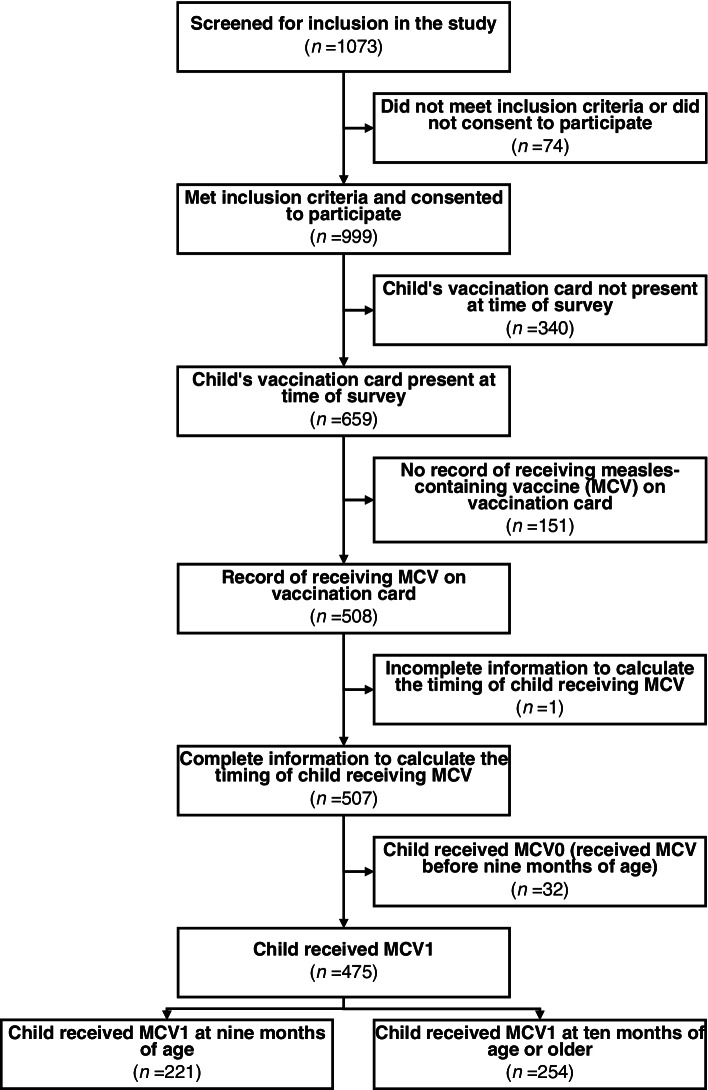
Study participants eligibility, availability of index children's vaccination cards, and the timing of index children receiving measles vaccination (MCV)

In total, 1073 eligible individuals were approached for study inclusion, and 999 (93.0%) completed the survey (Fig. 3). Participants ranged in age from 17 to 50 years, with a median of 27 years. The most commonly reported tribe was *Baganda* (singular: *Muganda*) (53.3%, *n* = 532) and highest level of education completed was secondary school (49.6%, *n* = 495). About one third of participants (35.2%, *n* = 352) were Catholic and about half of participants (55.7%, *n* = 556) reported not being employed outside the home. Approximately one quarter (23.0%, *n* = 230) of participants reported having one living child, and a similar proportion (27.5%, *n* = 275) reported having two living children (Table 1). The majority (77.2%, *n* = 771) of participants reported being currently married or living together with the index child’s father.

**Table 1:** Characteristics of survey participants (mothers/caregivers) overall, and by achievement of on-time measles vaccination (MCV1) for the index child

	Total (*n* = 999)			Among children with a vaccination record who were vaccinated with MCV1 on-time or delayed (*n* = 475)			
	n	%^a^	95% CI	Delayed (*n* = 254)		On-time (*n* = 221)	
				n	%^a^	n	%^a^
**Age (years)**							
Under 20	37	3.7	2.7, 5.1	8	3.2	3	1.4
20–24	267	26.7	24.1, 29.6	59	23.2	78	35.3
25–29	345	34.5	31.6, 37.5	92	36.2	78	35.3
30–34	185	18.5	16.2, 21.1	48	18.9	31	14.0
35+	165	16.5	14.3, 19.0	47	18.5	31	14.0
**Age (years) (Median [Range])**	27 [17,50]			28 [17,43]		26 [19,45]	
**Number of living children**							
1	230	23.0	20.5, 25.7	52	20.5	68	30.8
2	275	27.5	24.8, 30.4	69	27.2	67	30.3
3	223	22.3	19.8, 25.0	68	26.8	42	19.0
4+	271	27.1	24.4, 30.0	65	25.6	44	19.9
**Number of living children (Median [Range])**	2 [1,13]	2.8	[1,13]	3 [1,9]		2 [1,8]	
**Tribe**							
Muganda	532	53.3	50.1, 56.3	132	52.0	122	55.2
Muyankole	138	13.8	11.8, 16.1	36	14.2	30	13.6
Other	328	32.8	30.0, 35.8	86	33.9	69	31.2
**Missing**	1	0.1					
**Highest level of education completed**							
Did not attend/do not know	34	3.4	2.4, 4.7	9	3.5	2	0.9
Primary	382	38.2	35.3, 41.3	96	37.8	69	31.2
Secondary	495	49.6	46.5, 52.7	126	49.6	125	56.6
Post-secondary	85	8.5	6.9, 10.4	23	9.1	24	10.9
Missing	3	0.3		0	0.0	1	0.5
**Religion**							
Catholic	352	35.2	32.3, 38.3	91	35.8	85	38.5
Anglican	232	23.2	20.7, 25.9	62	24.4	45	20.4
Muslim	217	21.7	19.3, 24.4	43	16.9	49	22.2
Other religion	189	18.9	16.6, 21.5	55	21.7	39	17.7
Missing	9	0.9		3	1.2	3	1.4
**Relationship status with index child’s father**							
Currently married or living together	771	77.2	74.5, 79.7	203	79.9	183	82.8
Never married and never living together	88	8.8	7.2, 10.7	21	8.3	16	7.2
Formerly married	138	13.8	11.8, 16.1	30	11.8	22	10.0
**Missing**	2	0.2					
**Employed outside the home**							
No	556	55.7	52.6, 58.7	130	51.2	139	62.9
Yes	442	44.2	41.2, 47.3	124	48.8	82	37.1
Missing	1	0.1					

^a^Percentages may not equal 100 because of rounding

*Abbreviations*: *CI* Confidence Interval

The age of index children ranged from 12 to 72 months, with a median of 29 months (2.4 years). Slightly over half (52.8%, *n* = 527) of the children were male, and about one third (30.3%, *n* = 304) were the first-born child. Only 24 (2.4%) children were part of a multiple birth (Table 2).

**Table 2:** Characteristics of the index children overall, and by achievement of on-time measles vaccination (MCV1)

	Total (*n* = 999)			Among children with a vaccination record who were vaccinated with MCV1 on-time or delayed (*n* = 475)			
	n	%^a^	95% CI	Delayed (*n* = 254)		On-time (*n* = 221)	
				n	%^a^	n	%^a^
**Age (months)**							
12–23	354	35.4	32.5, 38.5	95	37.4	91	41.2
24–35	264	26.4	23.8, 29.3	67	26.4	54	24.4
36–47	174	17.4	15.2, 19.9	40	15.8	44	19.9
48–59	119	11.9	10.0, 14.1	31	12.2	19	8.6
60+	71	7.1	5.7, 8.9	21	8.3	10	4.5
Missing	17	1.7				3	1.4
**Age (months) (Median [Range])**	29 [12,72]			28 [12,71]		2, [12,70]	
**Sex**							
Female	470	47.1	44.0, 50.2	105	41.3	104	47.1
Male	527	52.8	49.6, 55.8	149	58.7	117	52.9
Missing	2	0.2					
**Birth order**							
First	304	30.4	27.7, 33.4	73	28.7	96	43.4
Second	253	25.3	22.7, 28.1	61	24.0	46	20.8
Third or higher	442	44.2	41.2, 47.3	120	47.2	79	35.8
**Part of a multiple birth**							
No	971	97.2	96.0, 98.1	245	96.5	212	95.9
Yes	24	2.4	1.6, 3.6	9	3.5	7	3.2
Missing	4	0.4				2	0.9

^a^Percent totals may not equal 100 due to rounding

*Abbreviations*: *CI* Confidence Interval

The majority of participants (71.1%, *n* = 710) reported giving birth to the index child in a public hospital/clinic. Most participants reported having completed the Uganda Ministry of Health-recommended number of four antenatal care visits during their pregnancy, with 40.0% (*n* = 400) reporting four visits and 34.9% (*n* = 349) reporting more than four. When asked who makes decisions about medical care for the index child, most (66.7%, *n* = 666) participants reported joint decision making with their spouses, while 18.5% (*n* = 185) said that they make the decisions on their own (Table 3).

**Table 3:** Healthcare characteristics of participants and index children overall, and by achievement of on-time measles vaccination (MCV1) for the index child

	Total (*n* = 999)			Among children with a vaccination record who were vaccinated with MCV1 on-time or delayed (n = 475)			
	n	%^a^	95% CI	Delayed (*n* = 254)		On-time (*n* = 221)	
				n	%^a^	n	%^a^
**Location of birth of index child**							
Public hospital/clinic	710	71.1	68.2, 73.8	194	76.4	153	69.2
Private hospital	230	23.0	20.5, 25.7	51	20.1	59	26.7
At home	56	5.6	4.3, 7.2	9	3.5	9	4.1
Missing	3	0.3					
**Number of antenatal care visits**							
No visits	12	1.2	0.7, 2.1	2	0.8	1	0.5
Less than four	235	23.5	21.0, 26.3	65	25.6	49	22.2
Four visits	400	40.0	37.0, 43.1	85	33.5	96	43.4
More than four visits	349	34.9	32.0, 37.9	102	40.2	75	33.9
Missing	3	0.3					
**Who makes medical care decisions for the index child?**							
Mother/caregiver alone	185	18.5	16.2, 21.1	43	16.9	34	15.4
Mother/caregiver and spouse	666	66.7	63.7, 69.5	181	71.3	159	72.0
Spouse alone	64	6.4	5.0, 8.1	13	5.1	10	4.5
Other	79	7.9	6.4, 9.8	16	6.3	17	7.7
Nobody does/blank/missing	5	0.5		1	0.4	1	0.5
**Moved to Rubaga in index child’s lifetime**							
No	755	77.0	74.3, 79.5	196	77.2	183	82.8
Yes	225	22.5	20.0, 25.2	58	22.8	34	15.4
Missing	19	0.5				4	1.8

^a^Percentage may not equal 100 due to rounding

*Abbreviations*: *CI* Confidence Interval

### Achievement of on-time MCV1 vaccination

Among all 999 index children, 50.9% (*n* = 508) had documentation that they were vaccinated with MCV, 15.1% (*n* = 151) had documentation that they were not vaccinated with MCV (presented a vaccination card with no date of measles vaccination listed), and 34.0% (*n* = 340) had no documentation at all and thus had unknown vaccination status.

**Fig. 4:**
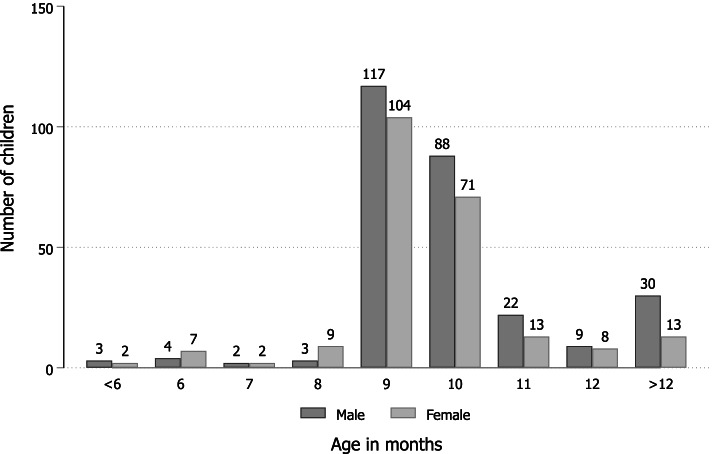
Distribution of index child’s age in months at the time of receiving MCV vaccination (*n* = 507)

Among the 508 index children who had documentation that they were vaccinated with MCV, 43.5% (*n* = 221) had documentation that they were vaccinated with MCV1on-time, 50.0% (*n* = 254) had documentation that their MCV1 vaccination was delayed, 6.3% (*n* = 32) had documentation that they were vaccinated early (vaccinated with MCV0), and one index child was missing information to calculate timing of MCV vaccination (0.2%) (Figs. 3, 4).

Of the 475 index children with known MCV1 vaccination status who were vaccinated on time or delayed, less than half 46.5% (*n* = 221) were vaccinated on-time and 53.5% (*n* = 254) were delayed, which was not significantly different from the hypothesized proportion of 50% (one-sample test of equality of proportions *p*-value = 0.13).

### Factors associated with achievement of on-time MCV1 vaccination

There was no association between a participant being able to identify information on their child’s vaccination card and achieving on-time MCV1vaccination in the univariate analysis, or after adjusting for healthcare and demographic factors in the multivariable analysis (Table 4).

**Table 4:** Logistic regression models to evaluate the association between ability to identify all three pieces of information (index child’s sex, date of birth, and MCV1 receipt information) on their vaccination card and achieving on-time MCV1 vaccination. cOR and aOR plus 95% CIs for the estimates obtained for univariate and multivariable logistics regression models are reported, respectively

	Univariate models (*n* = 469)			Multivariable model (*n* = 340)		
	cOR	95%CI	*p*-value	aOR	95% CI	*p*-value
**Mother/caregiver was able to identify key pieces of information on index child’s vaccination card**						
No	1.0	–	–	1.0	–	–
Yes	0.9	0.6, 1.4	0.75	0.7	0.5, 1.1	0.18
**Mother/caregiver age (years)**						
Under 20	0.4	0.1, 1.7	0.24	0.1	0.01, 1.0	0.05
20–24	1.6	1.0, 2.5	0.06	1.2	0.7, 2.1	0.52
25–29	1.0	–	–	1.0	–	–
30–34	0.8	0.4, 1.3	0.33	0.8	0.5, 1.5	0.57
35+	0.8	0.5, 1.3	0.37	1.2	0.6, 2.3	0.54
**Mother/caregiver employed outside the home**						
No	1.0	–	–	1.0	–	–
Yes	0.6	0.4, 0.9	0.01	0.7	0.4, 1.0	0.06
**Mother/caregiver highest level of education completed**						
Did not attend/do not know	0.3	0.1, 1.5	0.14	0.4	0.08, 2.0	0.26
Primary	1.0	–	–	1.0	–	–
Secondary	1.4	0.9, 2.1	0.11	1.4	0.9, 2.3	0.13
Post-secondary	1.5	0.8, 2.8	0.26	1.7	0.8, 3.7	0.18
**Index child birth order**						
First	1.0	–	–	1.0	–	–
Second	0.6	0.4, 0.9	0.03	0.6	0.3, 1.0	0.07
Third or higher	0.5	0.3, 0.8	0.01	0.6	0.3, 1.0	0.05
**Index child age (months)**						
12–23	1.0	–	–	1.0	–	–
24–35	0.8	0.5, 1.3	0.46	0.9	0.5, 1.5	0.73
36–47	1.1	0.7, 1.9	0.60	1.2	0.7, 2.1	0.51
48–59	0.6	0.3, 1.2	0.17	0.8	0.4, 1.6	0.47
60+	0.5	0.2, 1.1	0.09	0.5	0.2, 1.1	0.08
**Index child sex**						
Female	1.0	–	–	–	–	–
Male	0.8	0.6, 1.1	0.21	0.8	0.5, 1.2	0.30
**Who makes medical care decisions for the index child?**						
Mother/caregiver alone	1.0	–	–	–	–	–
Mother/caregiver and spouse	1.1	0.7, 1.8	0.68	–	–	–
Spouse alone	1.0	0.4, 2.5	0.95	–	–	–
Other	1.3	0.6, 3.0	0.48	–	–	–
**Relationship to index child’s father**						
Currently married or living together	1.0	–	–	–	–	–
Never married and never living together	0.8	0.4, 1.7	0.63	–	–	–
Formerly married	0.8	0.5, 1.5	0.49	–	–	–

*Abbreviations*: *cOR* Crude Odds ratio, *aOR* Adjusted Odds ratio, *CI* Confidence Interval

### Factors associated with ability to identify information on the vaccination card

**Fig. 5:**
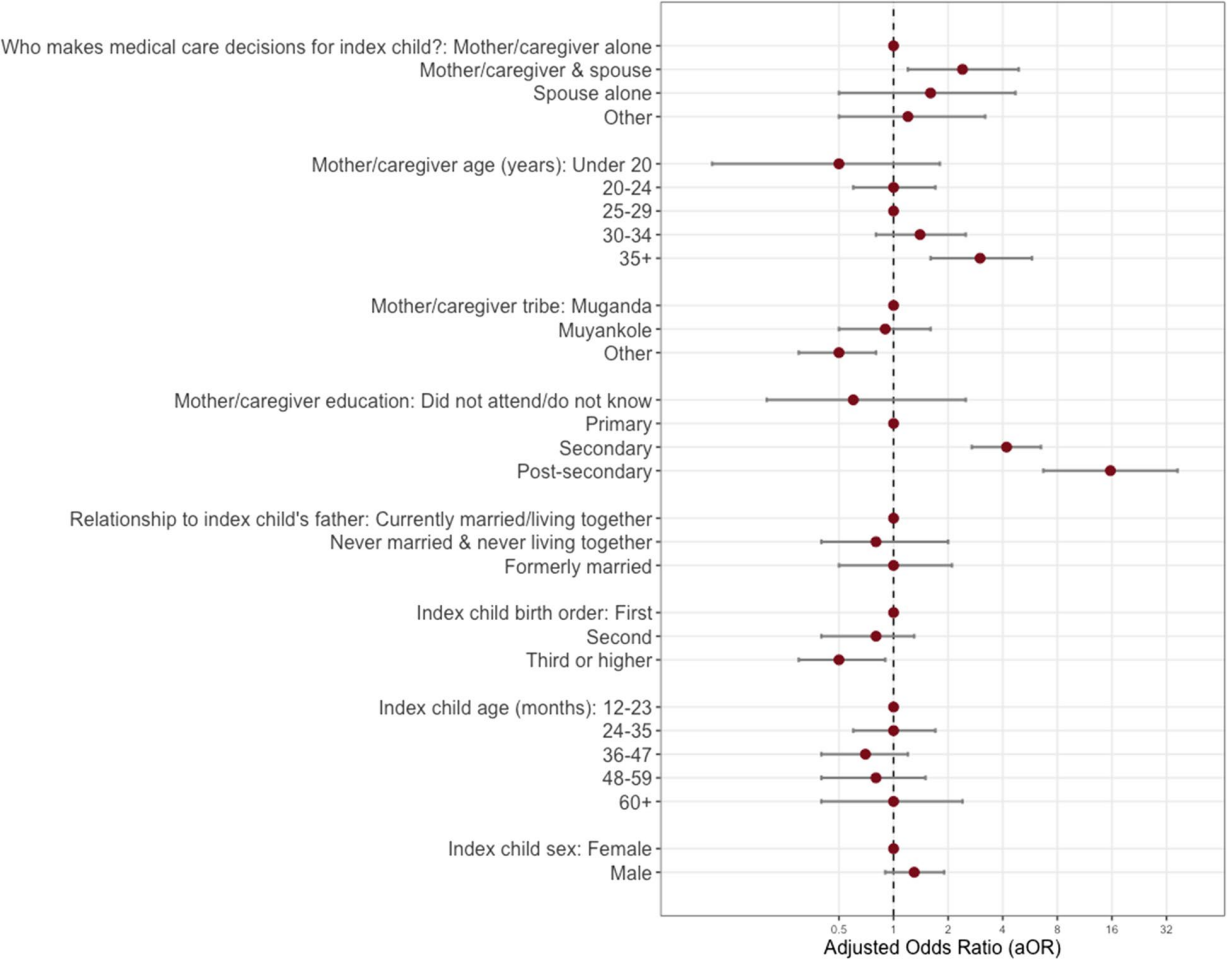
Multivariable logistic regression model to assess the factors associated with participant’s ability to identify all three key pieces of information (index child’s sex, date of birth, and MCV1 information), compared to identifying less than three or none (*n* = 542)

Of the 659 participants who had their child’s vaccination card present at the time of the survey, 551 answered all three questions about identifying information on the card. Of those, about half (47.9%, *n* = 264) could identify or point to all three pieces of information on the document: child’s date of birth, child’s sex, and child’s MCV1 information (Items A, B, and C of Fig. 2). We found that mothers/caregivers who were part of other tribes (compared to Muganda [aOR = 0.5; 95%CI:0.3, 0.8]) and children who were thirdborn or higher in the birth order (compared to the firstborn, [aOR = 0.5; 95%CI:0.3, 0.9]) had lower odds of being able to identify the information on the vaccination card. Compared to participants who reported completing primary education, those who reported completing secondary education [aOR = 4.2; 95%CI:2.7,6.5] or post-secondary education [aOR = 15.7; 95%CI:6.7,36.8]), and those who reported that medical decisions for the index child were made jointly with their spouse (compared to making medical decisions on her own [aOR = 2.4; 95%CI:1.2, 4.9]) had a higher odds of being able to identify the information on the vaccination card (Fig. 5).

### Factors independently associated with retention of child’s vaccination card

**Fig. 6:**
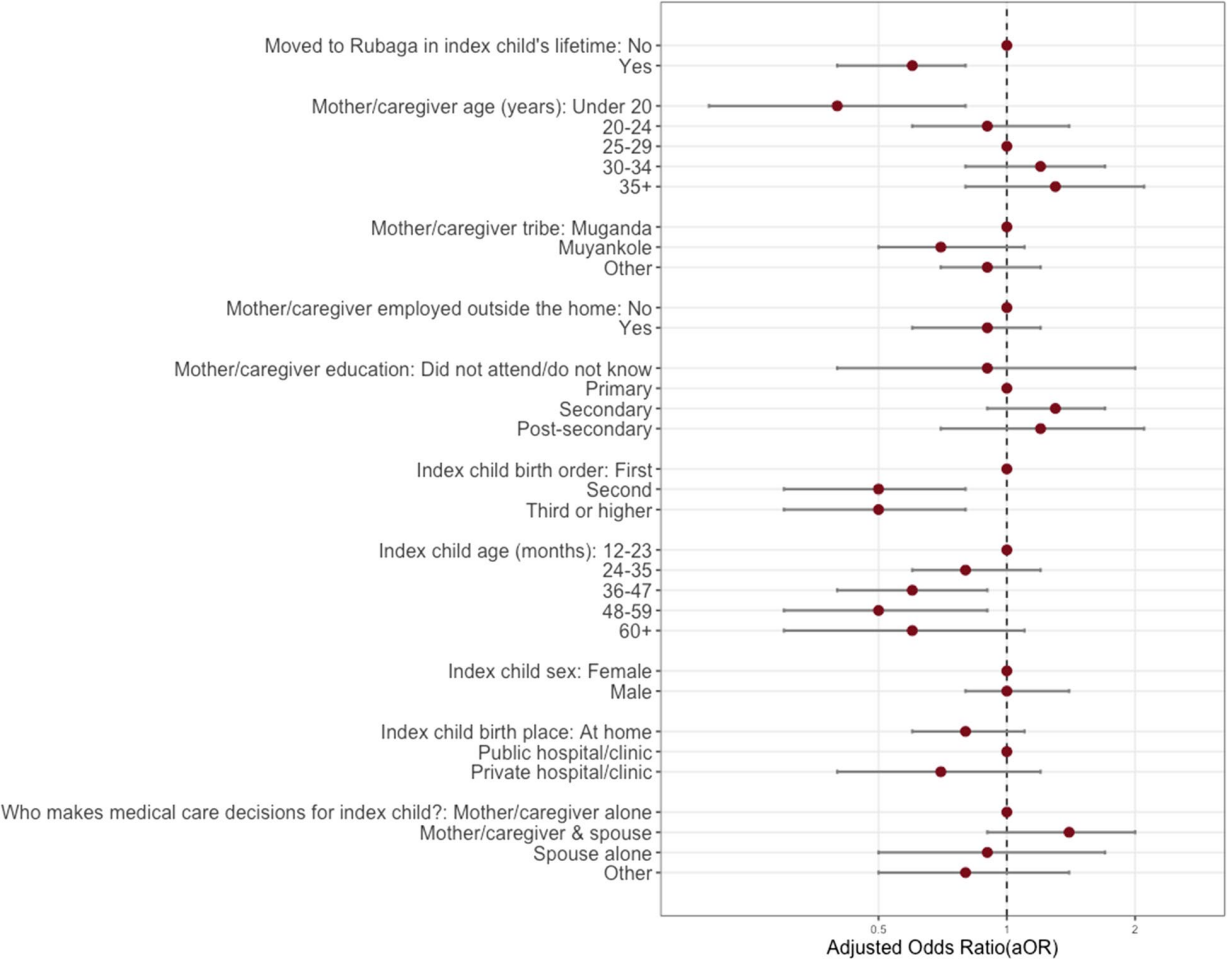
Multivariable logistic regression model to assess the factors associated with retention of the index child's vaccination card at the time of the survey (vs. did not retain vaccination card) (*n* = 973)

We found that participants who retained their index child’s vaccination card were significantly different from those who did not on certain characteristics, including whether they had moved into the district during the index child’s lifetime, child’s age, and the child’s birth order (Fig. 6). Participants who were under 20 years of age, compared to those who were 25 to 29 years of age, had lower odds of having their child’s vaccination card [aOR = 0.4; 95%CI:0.2, 0.8]. Compared to firstborn children, children higher in the birth order had a lower odds of having their child’s vaccination card (second born [aOR = 0.5; 95%CI:0.3, 0.8], third born or higher [aOR = 0.5; 95%CI:0.3, 0.8]), and those whose index child was 36–47 months of age [aOR = 0.6; 95%CI: 0.4, 0.9] or 48–59 months of age [aOR = 0.5; 95%CI:0.3, 0.9] (compared to children who were 12–23 months of age) had a lower odds of having their vaccination card. Additionally, participants who reported moving to Rubaga Division within the child’s lifetime, compared to participants who did not move during the child’s lifetime [aOR = 0.6; 95%CI:0.4, 0.8] had a lower odds of retaining their vaccination card (Fig. 6).

## Discussion

The primary aims in this study were to assess the proportion of children who were vaccinated with MCV1 on-time and delayed, and investigate the association between demographic factors, ability to identify information on the child’s vaccination card, and achieving on-time MCV1 vaccination. Our secondary aims were to investigate the association between demographic and healthcare factors and being able to identify key pieces of information on the vaccination card (vs. not being able to) and to investigate the association between demographic and healthcare factors and retaining the vaccination card (vs. not retaining). We found that over half of all participants were able to present some type of documentation of their child’s vaccination at the time of the survey.

Although 61.2% of the study population who had their child’s vaccination card at the time of the survey reported completing a secondary or post-secondary level of education, over half of participants with a vaccination card were not able to locate key pieces of information on the card that may help mothers or caregivers determine when their child was to be vaccinated for MCV1. Among participants who retained their child’s vaccination card, their ability to identify key pieces of information on the card, including the child’s measles vaccination information, is not independently associated with on-time vaccination, after accounting for multiple demographic and healthcare factors. Due to the structure of the study, we only explored these factors among a sample of vaccinated children. It is possible that factors such as retaining the vaccination card and being able to identify information on it may have a greater impact on children getting vaccinated, rather than achieving on-time vaccination. It is also possible that there are other factors beyond card retention and utilization that influence achiecement of on-time vaccination.

Among participants with information on the date of their child receiving MCV1, less than half achieved on-time vaccination, and over half were delayed. Our findings differ from a 2012 study conducted by Babirye et al. in a similarly aged, urban population in Uganda that found about two-thirds of children received vaccination within the recommended age. The difference may have arisen because that study defined on-time vaccination as occurring between 38 weeks to 12 months, whereas we defined on-time vaccination as receiving MCV1 in the ninth month of age.  Despite the broader time frame for achieving on-time vaccination, the authors noted that on-time measles vaccination was lower than any of the other recommended childhood vaccinations [16].

The purpose of the Uganda Ministry of Health Child Health Card is to monitor multiple aspects of a child’s health and growth, including to indicate the age at which a child should get a vaccination and the type of vaccine, to mothers/caregivers and healthcare workers. The vaccination card plays an important role in monitoring a child’s health, and hence the importance of retaining these records. We found that over half of study participants retained the card and presented it at the time of the survey. Not surprisingly, the majority of children with retained vaccination cards were born in either a public or private health facility. The proportion of participants who retained their child's vaccination card at the time of the survey is similar to the proportion of children aged 0 to 24 months with vaccination cards in a study in a similar setting [21]. That study also found that children delivered at a health facility were four times as likely to have a vaccination card, compared to those that were delivered at home. Another study conducted in a similarly aged population (0 to 24 months) in Nepal found that overall retention of the vaccination card was higher (82.2%) than in our study sample. In the Nepal study, vaccination card retention was 90.3% among 0–12 months children age group and 74% among children aged 12 to 24 months, indicating that child’s age may have an influence on retention of the card [31].

Relatedly, we found that the child’s birth order was independently associated with retention of the child’s vaccination card, with the odds of a participant retaining their child’s vaccination card being lower for children second-born or higher in the birth order, compared to firstborn. Furthermore, our univariate analyses revealed a strong association between birth order and achieving on-time vaccination, with children higher in the birth order having a lower odds of being vaccinated on time, compared to firstborn children. These findings are consistent with the findings of a study conducted in a similar population in Uganda, in which vaccinations that were not received during the recommended timeframe were associated with a higher number of children per woman (adjusted hazard ratio (AHR) 1.84, 95% CI 1.29, 2.64) [16, 32].

While about one third of the children in the study sample were one to two years of age, the majority were older than two years. This gap of time, which ranged from three months up to four years and three months between use of the card for vaccination and this survey may have influenced both the probability of the participants retaining the child’s vaccination card and the way participants responded to prompts about finding information on the card. Although this is the case, parents are instructed to retain their child’s vaccination card for documentation of routine vitamin A supplementation, deworming activities, and growth monitoring through five years of age [33].

We found that mother/caregiver-reported completion of secondary or post-secondary education, compared to primary education, was independently associated with their ability to identify information on their child’s vaccination card, including their child’s measles vaccination information, which may have influenced the timing of their child’s vaccinations.

In previous studies, mother’s education level was found to be a predictor of vaccination timing in multiple settings, with lower educational status associated with delayed vaccination in a study in Senegal, which used Demographic and Health Survey (DHS) data [34], and in other population-based surveys in Ghana [35] and Kenya [18]. Our analysis did not yield a similar finding, which may be due to the moderately high level of education in the study sample overall (49.7% of participants reported completing secondary education or higher).

The strengths of this study include a relatively large sample size within a population that is at an increased risk for measles infection. The clear aims of the study, along with the use of the child’s vaccination card increase the accuracy of the measured outcome of vaccination timeliness. Assessing the timing of the measles vaccination, in addition to whether the vaccine was received, increases opportunities to identify gaps in care and to advance research to improve vaccination timing and therefore increase protection from measles infection. Furthermore, the assessment of the vaccination card as a reminder tool for timely vaccination generates important new questions about how these cards are utilized and how their value can be increased.

The findings should be interpreted in light of a few limitations. First, vaccination timing was based on documented information on the child’s vaccination card, and only a subset of the surveyed population had a vaccination card. The significant difference between participants who retained and did not retain the card may have introduced selection bias into the sample, which limits the generalizability of the findings. Furthermore, for pragmatic reasons, the survey was only available in two languages: Luganda and English, which again introduced selection bias. This may not substantially limit the generalizability of the sample to other groups, because English is the national language of Uganda and Luganda is one of the most widely spoken languages. Third, this was a study of mothers/caregivers with surviving children who have not reached their sixth birthday and hence might have a survivor bias, as children who did not survive to this age might have been more likely to be under-vaccinated.

Finally, this study collected the mothers'/caregivers' self-report of the month and year of the child’s birth, thus it is not possible to assess the timing of the MCV1 dose to the day. Rather, this was estimated to the month. We expect some misclassification of age at measles vaccination by one month if the day of birth is greater than the day of survey administration. Therefore, there is also misclassification of on-time MCV1 vaccination status, based on when within the month the child was born. We consider this misclassification to be nondifferential, as the day of survey administration varied throughout the months that the data collection was taking place. Collecting date of birth information from the vaccination card itself may have improved the accuracy of child age calculations and reduced misclassification, although that information would be missing from the about one-third of children who did not have a vaccination card available at the time of the survey.

Nevertheless, these study findings are important for understanding the complex factors that are associated with achieving on-time measles vaccination, especially within a population at a high risk for measles infection.

## Conclusion

Being able to identify information on their child’s vaccination card was not associated with achieving on-time measles vaccination. New strategies are needed to both ensure that mothers/caregivers understand and can access the information on their child’s vaccination card, as this is the only documentation that indicates the age at which a child is due for MCV1 and is the only documentation that parents have of vaccine receipt. Further research can shed light on mechanisms by which measles vaccination is delayed and investigate factors that may prompt or remind mothers and other primary caregivers of the time when their child is due for a measles vaccine.

## Supplementary Information

**Additional file 1.**

## Data Availability

The datasets used and/or analysed during the current study are available from the corresponding author on reasonable request.

## Abbreviations

AFRO: WHO African Region
AHR: Adjusted Hazard Ratio
aOR: Adjusted odds ratio
AU: Administrative unit
CI: Confidence interval
cOR: Crude odds ratio
DHS: Demographic and Health Survey
DTwPHibHepB: Diphtheria and tetanus and pertussis and *Hemophilus influenzae* and hepatitis B vaccine
IPV: Inactivated polio vaccine
LMICs: Low- and Middle- Income Countries
MCV/MCV0/MCV1: Measles-containing vaccine/Measles-containing vaccine, dose 0/Measles-containing vaccine, dose 1
MeV: *Measles morbillivirus*
PCV: Pneumococcal conjugate vaccine
SOMREC: Makerere University School of Medicine Research and Ethics Committee
UCHC: Uganda Ministry of Health Child Health Card
UNCST: Uganda National Council for Science and Technology
UNEPI: Uganda National Expanded Program on Immunisation
UNICEF: United Nations Children’s Fund
WHO: World Health Organization
