# Supplementary material for: Does mothers’ and caregivers' access to information on their child’s vaccination card impact the timing of their child’s measles vaccination in Uganda?
Source: BMC Public Health. 2022 Apr 26;22:834. doi: 10.1186/s12889-022-13113-z (PMC9044684; doi:10.1186/s12889-022-13113-z)
Supplement: Supplementary file 1 — Additional file 1. [file 12889_2022_13113_MOESM1_ESM.doc]

| Supplemental table 1: Logistic regression models to evaluate the factors associated with retention of the vaccination card at the time of the survey (vs. no document) | | | | | | | |
| --- | --- | --- | --- | --- | --- | --- | --- |
|  |  | Univariate models (n=999) | | | Multivariable model (n=973)* | | |
|  |  | cOR | 95% CI | p-value | AOR | 95% CI | p-value |
| **Moved to Rubaga Division in the index child’s lifetime** | No | 1.0 | -- | -- | 1.0 | -- | -- |
|  | Yes | 0.5 | 0.4, 0.7 | 0.00 | 0.6 | 0.4, 0.8 | 0.00 |
|  |  |  |  |  |  |  |  |
| **Mother/caretaker’s age (years)** | Under 20 | 0.7 | 0.4, 1.4 | 0.33 | 0.4 | 0.2, 0.8 | 0.02 |
|  | 20-24 | 1.3 | 0.9, 1.8 | 0.15 | 0.9 | 0.6, 1.4 | 0.78 |
|  | 25-29 | 1.0 | -- | -- | 1.0 | -- | -- |
|  | 30-34 | 0.9 | 0.6, 1.3 | 0.70 | 1.2 | 0.8, 1.7 | 0.47 |
|  | 35+ | 1.1 | 0.7, 1.6 | 0.80 | 1.3 | 0.8, 2.0 | 0.22 |
|  |  |  |  |  |  |  |  |
| **Tribe** | Muganda | 1.0 | -- | -- | 1.0 | -- | -- |
|  | Muyankole | 0.7 | 0.5, 1.0 | 0.04 | 0.7 | 0.5, 1.1 | 0.09 |
|  | Other | 0.9 | 0.7, 1.2 | 0.61 | 0.9 | 0.7, 1.2 | 0.50 |
|  |  |  |  |  |  |  |  |
| **Mother/caretaker employed outside the home** | No | 1.0 | -- | -- | 1.0 | -- | -- |
|  | Yes | 0.8 | 0.6, 1.1 | 0.19 | 0.9 | 0.6, 1.2 | 0.33 |
|  |  |  |  |  |  |  |  |
| **Highest level of education** | Did not attend/do not know | 0.9 | 0.4, 1.8 | 0.76 | 0.9 | 0.4, 2.0 | 0.88 |
|  | Primary | 1.0 | -- | -- | 1.0 | -- | -- |
|  | Secondary | 1.4 | 1.1, 1.9 | 0.01 | 1.3 | 0.9, 1.7 | 0.12 |
|  | Post-secondary | 1.4 | 0.9, 2.4 | 0.17 | 1.2 | 0.7, 2.1 | 0.51 |
|  |  |  |  |  |  |  |  |
| **Index child’s birth order** | First | 1.0 | -- | -- | 1.0 | -- | -- |
|  | Second | 0.6 | 0.4, 0.9 | 0.01 | 0.5 | 0.3, 0.8 | 0.00 |
|  | Third or higher | 0.7 | 0.5, 0.9 | 0.01 | 0.5 | 0.3, 0.8 | 0.00 |
|  |  |  |  |  |  |  |  |
| **Age (months)** | 12-23 | 1.0 | -- | -- | 1.0 | -- | -- |
|  | 24-35 | 0.8 | 0.6, 1.2 | 0.27 | 0.8 | 0.6, 1.1 | 0.23 |
|  | 36-47 | 0.6 | 0.4, 0.9 | 0.01 | 0.6 | 0.4, 0.9 | 0.02 |
|  | 48-59 | 0.5 | 0.3, 0.8 | 0.00 | 0.5 | 0.3, 0.9 | 0.01 |
|  | 60+ | 0.6 | 0.3, 0.9 | 0.03 | 0.6 | 0.3, 1.1 | 0.13 |
|  |  |  |  |  |  |  |  |
| **Index child sex** | Female | 1.0 | -- | -- | 1.0 | -- | -- |
|  | Male | 1.0 | 0.7, 1.3 | 0.8 | 1.0 | 0.8, 1.4 | 0.28 |
|  |  |  |  |  |  |  |  |
| **Location of index child’s birth** | Public hospital/clinic | 1.0 | -- | -- | 1.0 | -- | -- |
|  | Private hospital/clinic | 0.8 | 0.6, 1.1 | 0.13 | 0.8 | 0.6, 1.1 | 0.14 |
|  | At home | 0.6 | 0.4, 1.1 | 0.10 | 0.7 | 0.4, 1.2 | 0.19 |
|  |  |  |  |  |  |  |  |
| **Who makes decisions about medical care for the index child?** | Mother/caretaker alone | 1.0 | -- | -- | 1.0 | -- | -- |
|  | Mother/caretaker and partner | 1.6 | 1.1, 2.2 | 0.01 | 1.4 | 0.9, 2.0 | 0.06 |
|  | Partner alone | 0.9 | 0.5, 1.6 | 0.65 | 0.9 | 0.5, 1.7 | 0.84 |
|  | Other | 1.0 | 0.6, 1.6 | 0.85 | 0.8 | 0.5, 1.5 | 0.51 |
|  |  |  |  |  |  |  |  |
| **Relationship to index child’s father** | Currently married or living together | 1.0 | -- | -- | -- | -- | -- |
|  | Never married and never living together | 1.0 | 0.6, 1.6 | 0.99 | -- | -- | -- |
|  | Formerly married | 0.5 | 0.4, 0.8 | 0.001 | -- | -- | -- |
|  |  |  |  |  |  |  |  |
| **Religion** | Catholic | 1.0 | -- | -- | -- | -- | -- |
|  | Anglican | 0.8 | 0.6, 1.2 | 0.27 | -- | -- | -- |
|  | Muslim | 0.9 | 0.6, 1.3 | 0.49 | -- | -- | -- |
|  | Other | 1.1 | 0.7, 1.6 | 0.73 | -- | -- | -- |

**Abbreviations: Crude Odds ratio (cOR); Adjusted Odds ratio (AOR); Confidence Interval (CI)**

***Participants with non-missing information were included in the multivariable model.**

| Supplemental table 2: Logistic regression models to assess the factors associated with participant’s ability to identify all three pieces of information (index child’s sex, date of birth, and MCV1 receipt information), compared to locating less than three or none | | | | | | | |
| --- | --- | --- | --- | --- | --- | --- | --- |
|  |  | Univariate models (n=551) | | | Multivariable model (n=542) | | |
|  |  | cOR | 95% CI | p-value | AOR | 95% CI | p-value |
| **Who makes decisions about medical care for the index child?** | Mother/caretaker alone | 1.0 | -- | -- | 1.0 | -- | -- |
|  | Mother/caretaker and partner | 2.2 | 1.4, 3.5 | 0.001 | 2.4 | 1.2, 4.9 | 0.01 |
|  | Partner alone | 1.1 | 0.5, 2.6 | 0.84 | 1.6 | 0.5, 4.7 | 0.41 |
|  | Other | 1.3 | 0.6, 2.9 | 0.55 | 1.2 | 0.5, 3.2 | 0.70 |
|  |  |  |  |  |  |  |  |
| **Mother/caretaker’s age (years)** | Under 20 | 0.6 | 0.2, 1.9 | 0.35 | 0.5 | 0.1, 1.8 | 0.27 |
|  | 20-24 | 1.1 | 0.7, 1.7 | 0.63 | 1.0 | 0.6, 1.6 | 0.99 |
|  | 25-29 | 1.0 | -- | -- | 1.0 | -- | -- |
|  | 30-34 | 0.9 | 0.5, 1.4 | 0.62 | 1.4 | 0.8, 2.5 | 0.27 |
|  | 35+ | 1.2 | 0.7, 1.9 | 0.56 | 3.0 | 1.6, 5.8 | 0.00 |
|  |  |  |  |  |  |  |  |
| **Tribe** | Muganda | 1.0 | -- | -- | 1.0 | -- | -- |
|  | Muyankole | 0.8 | 0.5, 1.3 | 0.40 | 0.9 | 0.5, 1.6 | 0.62 |
|  | Other | 0.5 | 0.4, 0.8 | 0.00 | 0.5 | 0.3, 0.8 | 0.00 |
|  |  |  |  |  |  |  |  |
| **Highest level of education** | Did not attend/do not know | 0.7 | 0.2, 2.4 | 0.53 | 0.6 | 0.2, 2.5 | 0.51 |
|  | Primary | 1.0 | -- | -- | 1.0 | -- | -- |
|  | Secondary | 4.1 | 2.7, 6.1 | 0.00 | 4.2 | 2.7, 6.5 | 0.00 |
|  | Post-secondary | 13.4 | 6.1, 29.4 | 0.00 | 15.7 | 6.7, 36.8 | 0.00 |
|  |  |  |  |  |  |  |  |
|  |  |  |  |  |  |  |  |
| **Relationship to index child’s father** | Currently married or living together | 1.0 | -- | -- | 1.0 | -- | -- |
|  | Never married and never living together | 0.6 | 0.3, 1.1 | 0.10 | 0.8 | 0.4, 2.0 | 0.69 |
|  | Formerly married | 0.7 | 0.4, 1.1 | 0.12 | 1.0 | 0.5, 2.2 | 0.96 |
|  |  |  |  |  |  |  |  |
| **Index child’s birth order** | First | 1.0 | -- | -- | 1.0 | -- | -- |
|  | Second | 0.7 | 0.4, 1.0 | 0.07 | 0.8 | 0.4, 1.3 | 0.34 |
|  | Third or higher | 0.6 | 0.4, 0.9 | 0.07 | 0.5 | 0.3, 0.9 | 0.01 |
|  |  |  |  |  |  |  |  |
| **Age (months)** | 12-23 | 1.0 | -- | -- | 1.0 | -- | -- |
|  | 24-35 | 1.0 | 0.7, 1.5 | 0.99 | 1.0 | 0.6, 1.7 | 0.87 |
|  | 36-47 | 0.8 | 0.5, 1.3 | 0.33 | 0.7 | 0.4, 1.2 | 0.22 |
|  | 48-59 | 0.9 | 0.5, 1.6 | 0.80 | 0.8 | 0.4, 1.5 | 0.46 |
|  | 60+ | 1.3 | 0.6, 2.8 | 0.43 | 1.0 | 0.4, 2.4 | 0.95 |
|  |  |  |  |  |  |  |  |
| **Index child sex** | Female | 1.0 | -- | -- | 1.0 | -- | -- |
|  | Male | 1.3 | 0.9, 1.8 | 0.14 | 1.3 | 0.9, 1.9 | 0.22 |
|  |  |  |  |  |  |  |  |
| **Religion** | Catholic | 1.0 | -- | -- | -- | -- | -- |
|  | Anglican | 0.9 | 0.6, 1.4 | 0.67 | -- | -- | -- |
|  | Muslim | 0.8 | 0.5, 1.2 | 0.27 | -- | -- | -- |
|  | Other | 1.1 | 0.7, 1.7 | 0.78 | -- | -- | -- |
|  |  |  |  |  |  |  |  |
| **Mother/caretaker employed outside the home** | No | 1.0 | -- | -- | -- | -- | -- |
|  | Yes | 1.2 | 0.8, 1.6 | 0.34 | -- | -- | -- |
|  |  |  |  |  |  |  |  |

**Abbreviations: Crude Odds ratio (cOR); Adjusted Odds ratio (AOR); Confidence Interval (CI)**
